# EMTST: Engineering the Meta-theory of Session Types

**DOI:** 10.1007/978-3-030-45237-7_17

**Published:** 2020-03-13

**Authors:** David Castro, Francisco Ferreira, Nobuko Yoshida

**Affiliations:** 8 Johannes Kepler University, Linz, Austria; 9 University of Birmingham, Birmingham, UK; Imperial College London, London, UK

**Keywords:** Concurrency, proof assistants, meta-theory, session-types

## Abstract

Session types provide a principled programming discipline for structured interactions. They represent a wide spectrum of type-systems for concurrency. Their type safety is thus extremely important. EMTST is a tool to aid in representing and validating theorems about session types in the Coq proof assistant. On paper, these proofs are often tricky, and error prone. In proof assistants, they are typically long and difficult to prove. In this work, we propose a library that helps validate the theory of session types calculi in proof assistants. As a case study, we study two of the most used binary session types systems: we show the impossibility of representing the first system in $\alpha$-equivalent representations, and we prove type preservation for the revisited system. We develop our tool in the Coq proof assistant, using locally nameless for binders and small scale reflection to simplify the handling of linear typing environments.

## 1 Introduction

Given the prevalence of distributed computing and multi-core processors, concurrency is a key aspect of modern computing. The transition from sequential models of computation to concurrent systems has huge practical and theoretical consequences. Message passing calculi (like the $\pi$-calculus) have been used to model these systems since their introduction by Milner et al. [15]. Notably, in many cases *typing disciplines* are used as a way to control concurrent and distributed behaviour. Certifying basic typed $\pi$-calculi is important for both the safety of implementations and the trustworthiness of new theories.

In this work, we concentrate on providing tools for reasoning about *session types* [10], a typing discipline for structured interactions in distributed systems. Session types are applied to a wide range of problems, and their properties, such as deadlock-freedom, are well studied. These calculi are very expressive, and rather complex, with features like: shared and linear communication channels, name passing, and fresh name generation. Given this complexity, it is not surprising that some innocent looking extensions violated the type safety properties of the calculus in several literature (as pointed out by [23]). In consequence, the interest for mechanisation and formal proofs has risen significantly as a means to increase the trust on systems.

Type systems offer certain security properties by construction. These guarantees are backed by rigorous proofs (these proofs conform the meta-theory of the system). Moreover, these proofs are cumbersome to write, maintain and extend. Proof assistants aim to help with these problems. In this work, we develop the EMTST library to aid in the implementation of session calculi type systems. As a form of validation, we implement and replicate results in the meta-theory of binary session types. Concretely, we use the Coq proof assistant [20] to study the representation and meta-theory of the two systems described in [23].

EMTST uses *locally nameless* (LN)[1, 5] variable binders to represent syntax. The tool implements a LN library with extended support for multiple binding scopes, a robust environment implementation suitable for the challenges of session typing disciplines. The library and lemmas are written taking advantage of boolean reflection through the use of the `Ssreflect` [7] library.

We implement two case studies from [23]. The first study that we refer to as *the original system* and the second that we refer to as *the revised* systems. Notably, the way the original system handles names (in Sect. 3.1), makes its representation impossible when using intrinsically $\alpha$-convertible terms (e.g: locally nameless, de Bruijn indices, and many others). Furthermore in Sect. 3.2, we discuss how the revised system allows us to implement and prove type preservation. In hindsight, this problem appears as evident, but it is an unexpected consequence, and it shows that mechanising proofs brings further understanding even to well-established and thoroughly studied systems. EMTST and our case studies are available at https://github.com/emtst/emtst-proof.

The rest of the paper is structured in the following way: in the next section we introduce the ideas and design behind EMTST our library for mechanising the meta-theory of session types. Subsequently in Sect. 3, we present the two case studies: in Sect. 3.1 the original system from [23, 11] and the revisited system in Sect. 3.2. We finalise, by giving a conclusion and related work.

## 2 EMTST: a Tool for Representing the Meta-theory of Session Types

The study of meta-theory (i.e: proving a system has the expected properties) gives us confidence in the design. Additionally, proof formalisations, not only give us confidence in the results, but also often result in new insights about a problem. This is due to the fact that successful mechanisations require very precise specifications and careful thought to define and revisit all the concepts. In this context, EMTST is a tool that implements locally nameless (initially proposed by [8, 14, 13], and more recently further developed in [1, 5]) with multiple binding scopes, and a robust typing environment implementation using boolean reflection (by building on top of ssreflect [7]).

The key concept of LN is to use de Bruijn indices [2] for bound variables and names (sometimes called "atoms" in the literature) for free variables. A

representation of syntax is well formed, namely *locally closed*, when this invariant is respected (i.e.: no de Bruijn index is free). Finally, in order to deal with open terms, there are two convenient operations on syntax, one is to *open* binders in terms, and one to *close* binders. The former substitutes a bound variable with a fresh name, and the other does the converse. For more details, refer to our tech report [4], the references, and the implementation.

### 2.1 Environments and Multiple Name Scopes

```
Module Type ATOM.
  Parameter atom : Set.
  Definition t := atom.

(* atoms can be compared to booleans *)
  Parameter eq_atom : atom → atom → bool.
  Parameter eq_reflect : ∀(a b : atom),
    ssrbool.reflect (a = b) (eq_atom a b).
  Parameter atom_eqMixin : Equality.mixin_of atom.
  Canonical atom_eqType := EqType atom atom_eqMixin.

Parameter fresh : seq atom → atom.
  Parameter fresh_not_in : ∀l, (fresh l) ∉ l.
  (* ... *)
End ATOM.
```

**Figure 1.** The type of atoms

***Locally nameless implementation*** is in three files. The first (`theories/Atom.v`) provides the basic definition and specification of atoms to act as names, the second one (`theories/AtomScopes.v`) provides a way to create multiple disjoint sets of names for representing variables in the different scopes that session types require (e.g. variables and channel names), and the final one (`theories/Env.v`) implements contexts and typings as finite maps, with emphasis on supporting the linearity requirements of various session typing disciplines.

We use module types and parametrised modules to abstract the type of atoms together with their supported operations. Figure 1 shows the interface for working with atoms: how to compare them and functions to obtain a fresh atom given a finite sequence of atoms (definition: `fresh`), and to have proof that the fresh atom is actually fresh (definition: `fresh_not_in`).

***Environments.*** Environments are parametrised over two types, one for the keys, and one for the type of values. Environments `env` are either undefined, or a finite map of unique keys and values. All the operations keep the invariant that any operation that would lead to a duplicated entry key makes the tree undefined. We define the expected operations and lemmas over the type `env`. We provide an extensive library of proved theorems about environments that is tailored to support linear and affine systems.

EMTST is used in the two formalisations in Sect. 3.1 and 3.2 and we claim they are also suitable for other mechanisations where resource sensitivity and locally nameless are required. A release version of EMTST is available at [3] and the public repository at: https://github.com/emtst/emtst-proof.

## 3 Two Case Studies on Binary Session Types

EMTST is intended to help with the complex binding structure of concurrent calculi that have names as a first class notion together with linear or affine typing disciplines. We study two seminal session type systems in the literature. First the *original system*, from Honda, Vasconcelos and Kubo's binary session type system [11] that is a milestone in the development of type systems for concurrent process calculi. This system types structured interaction between processes and supports channel mobility, that is higher-order sessions. Second, we implement the revisited session type presentation from [23], inspired by [6]. Our technical report [4] contains an extensive presentation.

### 3.1 The Original System

Process $P, Q, R ::=$

| | | | |
|---|---|---|---|
| $\mid$ $\texttt{request}\ a\,(k).P$ | session request | $\mid$ $\texttt{if}\ e\ \texttt{then}\ P\ \texttt{else}\ Q$ | conditional |
| $\mid$ $\texttt{accept}\ a\,(k).P$ | session accept | $\mid$ $P \mid Q$ | parallel |
| $\mid$ $k\,![e];\ P$ | data send | $\mid$ $\texttt{inact}$ | inaction |
| $\mid$ $k\,?(x).P$ | data receive | $\mid$ $\nu_n\,(a).P$ | name hiding |
| $\mid$ $k \triangleleft m; P$ | selection | $\mid$ $\nu_c\,(k).P$ | channel hiding |
| $\mid$ $k \triangleright \{\texttt{l} : P[\texttt{r} : Q\}$ | branching | $\mid$ $!P$ | replication |
| $\mid$ $\texttt{throw}\ k\,[k'];P$ | channel send | | |
| $\mid$ $\texttt{catch}\ k\,(k').P$ | channel receive | | |
| $e ::= \texttt{true} \mid \texttt{false} \mid \ldots$ | expression | $m ::= \texttt{l} \mid \texttt{r}$ | labels |

**Figure 2.** Syntax using names

Figure 2 presents the syntax following [23], where names are ranged by $a$, $b$, $c$,..., channels are ranged by $k$ and $k'$. Notice that all the places where there are variable binders are denoted with parenthesis followed by a dot (e.g: $k\,?(x).P$). The syntax is straightforwardly defined as the `proc` inductive type in `theories/Syntax0.v` and following the LN technique the locally closed predicate, that formalises the binding structure, is defined as the predicate `lc`.

Besides its syntax, the original system is specified by its reduction, congruence and typing relations. We want to call attention to an important reduction rule for passing names:

$$[\textsc{Pass-Nm}] \qquad \texttt{throw}\ k\,[k'];P \mid \texttt{catch}\ k\,(k').Q \longrightarrow P \mid Q$$

This rule states that when passing a channel k'the receiving end has to bind a channel using the same name (or be $\alpha$-convertible to that name). Notoriously, the name k'is a bound name in the receiving end, and the restriction imposed by the rule is a subtle change to the equality up-to $\alpha$-conversion convention. Moreover, relaxations of that requirement may break subject reduction, a complete discussion is presented in Sect. 3 of [23]. As it is, this rule cannot be formalised in a representation that cannot distinguish between $\alpha$-equivalent terms. Since in these representations, one cannot talk about the actual name of a bound variable. This is fundamentally what it means to be *up-to $\alpha$-equality*. As a consequence, in locally nameless we are forced to specify the following rule:

$$[\textsc{Pass-LN}] \qquad \frac{\texttt{lc}\,P \qquad \texttt{body}\,Q}{\texttt{throw}\ k\,[k'];P \mid \texttt{catch}\ k\,().Q \longrightarrow P \mid Q^{k'}}$$

In this version of the rule, the bound name is just an anonymous de Bruijn index, and when it is opened it is assigned the same name k'. This change might look innocent, but it breaks subject reduction. In `theories/Types0.v`, we show that the same counter example from [23] is typable and that it breaks subject reduction. This is presented in the `CounterExample` module and in the `oft_reduced` lemma. In the next section, we discuss how this problem was addressed.

### 3.2 The Revised System

As discussed in Sect. 3.1 and [23], the presentation of the original session types calculus [11] makes extending it (and representing it in LN) a delicate operation. Fortunately, the revised system (also from [23], inspired by [6]) proposes a solution. Indeed, this solution is readily implementable using LN (and many other representations with implicit $\alpha$-equivalence).

The key insight in the design of the revisited system is considering *channel endpoints* instead of just *channels*. As before, a new channel is created when a requested session is accepted, and each continuation gets one of the *endpoints* of the newly created channel.

```
Inductive proc : Set :=
| request : scvar → proc → proc
| accept : scvar → proc → proc

| send : channel → exp → proc → proc
| receive : channel → proc → proc

| select :
  channel → label → proc → proc
| branch :
  channel → proc → proc → proc

| throw :
  channel → channel → proc → proc
| catch : channel → proc → proc

| ife : exp → proc → proc → proc
| par : proc → proc → proc
| inact : proc

| nu_ch : proc → proc (* hides a channel name *)
| nu_nm : proc → proc (* hides a name *)
| bang : proc → proc (* process replication *)
.
```

Legend:

| | |
|---|---|
| proc | process binds variable from $\mathbb{A}_{\texttt{SC}}$ |
| proc | process binds variable from $\mathbb{A}_{\texttt{EV}}$ |
| proc | process binds variable from $\mathbb{A}_{\texttt{LC}}$ |
| proc | process binds variable from $\mathbb{A}_{\texttt{CN}}$ |

**Figure 3.** Syntax representation annotated with binders

For the revisited system's formalisation we distinguish binders in four categories (as shown in Figure 3): First, expression variables, with names from the set $\mathbb{A}_{\texttt{EV}}$, then shared channel variables from $\mathbb{A}_{\texttt{SC}}$, also linear channel variables from $\mathbb{A}_{\texttt{LC}}$, and finally channel names from $\mathbb{A}_{\texttt{CN}}$ (these names can also be bound in restrictions). Channel names are not variables, but objects that exist at run-time.

Multiple disjoint sets of names simplify reasoning about free names (concretely, it avoids freshness problems among different kinds of binders). This is an engineering compromise, as having more binders duplicates some easy theorems but, in exchange, they simplify the harder theorems that rely on facts about LN open/close operations. Other compromises are possible.

This concludes the technical development, and represents a full proof of subject reduction for binary types, following the revised system[1] as defined in [23].

## 4 Related Work and Conclusions

We presented EMTST, a tool conceived to aid in the mechanisation of session calculi. Our tool supports locally nameless representations with many disjoint atom scopes, and a versatile representation of environments. All while taking advantage of the small scale reflection style of proofs. We validated our design by formalising the subject reduction proof for a full session calculus type system. And, we explored issues with adequacy when, for example, systems contain fragile specifications.

Tools like Metalib [22] (implemented based on [1]) and AutoSubst [18] exist, but lack the ability to represent different binding scopes in the same syntax. Also, Polonowski [17] implements a library for generic environments, while this library is similar to ours, it does not make use of boolean reflection, that, in our opinion simplifies dealing with the equality of environments. While these libraries were influential, our requirements of multiple scopes of binding and boolean reflection proofs, means that we needed to develop EMTST, our own fit for purpose library.

Finally, formalisations of session types in proof assistants exist in the literature (e.g.: [21, 24, 19, 16, 9]). Most of them with ad-hoc binder representations. They are not necessarily meant to be reused or general enough for other developments. This paper, and the EMTST library are a step towards helping this become easier. For that purpose we developed the library and validated its claims by formalising existing systems from the literature. In the process (see Sect. 3.1 vs Sect. 3.2), we motivate how early mechanisation would help avoid problems in the presentation of a system. In the future, we plan to extend our use of the library to reason about multiparty session types [12] and other systems.

## Acknowledgements

This work was supported in part by EPSRC projects EP/K011715/1, EP/K034413/1, EP/L00058X/1, EP/N027833/1, EP/N028201/1, and EP/T006544/1.

[1] A minor difference is that we use a simpler version of recursion compared to the original paper.
